# A Versatile Sulfur‐Assisted Pyrolysis Strategy for High‐Atom‐Economy Upcycling of Waste Plastics into High‐Value Carbon Materials

**DOI:** 10.1002/advs.202206924

**Published:** 2023-03-29

**Authors:** Youchen Tang, Zongheng Cen, Qian Ma, Bingna Zheng, Zhaopeng Cai, Shaohong Liu, Dingcai Wu

**Affiliations:** ^1^ Department of Orthopedics The Eighth Affiliated Hospital Sun Yat‐sen University Shenzhen 518000 P. R. China; ^2^ PCFM Lab School of Chemistry Sun Yat‐sen University Guangzhou 510006 P. R. China; ^3^ Research Center of Medical Sciences Guangdong Provincial People's Hospital Guangdong Academy of Medical Sciences Guangzhou 510080 P. R. China

**Keywords:** sodium‐ion batteries, sulfurization, upcycling, waste plastics

## Abstract

With the overconsumption of disposable plastics, there is a considerable emphasis on the recycling of waste plastics to relieve the environmental, economic, and health‐related consequences. Here, a sulfur‐assisted pyrolysis strategy is demonstrated for versatile upcycling of plastics into high‐value carbons with an ultrahigh carbon‐atom recovery (up to 85%). During the pyrolysis process, the inexpensive elemental sulfur molecules are covalently bonded with polymer chains, and then thermally stable intermediates are produced via dehydrogenation and crosslinking, thereby inhibiting the decomposition of plastics into volatile small hydrocarbons. In this manner, the carbon products obtained from real‐world waste plastics exhibit sulfur‐rich skeletons with an enlarged interlayer distance, and demonstrate superior sodium storage performance. It is believed that the present results offer a new solution to alleviate plastic pollution and reduce the carbon footprint of plastic industry.

## Graphical abstract

A sulfur‐assisted pyrolysis strategy is developed for versatile and high‐atom‐economy upcycling of the real‐world waste plastics into high‐value carbons, which have sulfur‐rich skeletons with an enlarged interlayer distance and thus demonstrate great application potential for high‐performance sodium‐ion batteries.

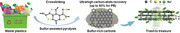

## 1 Introduction

As an important class of resilient and lightweight materials, plastics are indispensable in many facets of modern society, and have been wildly used in packaging, construction, transportation, electronics, and health‐care industries. Owing to their convenience and low cost, the demand for plastics has steadily increased, and global plastic production is predicted to reach 800 million tons per year by 2040.^[^ ^1^ ^]^ However, most of the plastics, such as polyethylene (PE), polypropylene (PP), poly(vinyl chloride) (PVC), and polystyrene (PS), are naturally non‐degradable and discarded after their short service life, resulting in significant accumulation in the natural environment and thereby giving rise to a serious threat to the ecosystem and human health.^[^ ^2^ ^]^ In addition, plastic production has a high carbon footprint, and the greenhouse gas emissions from plastics would reach 15% of the global carbon budget by 2050 if no efforts are made.^[^ ^3^ ^]^ Closed‐loop recycling of the post‐consumer plastics is a promising strategy to overcome the global plastic waste crisis and reduce the greenhouse gas emissions from plastics.^[^ ^4^ ^]^ Unfortunately, conventional mechanical recycling based on melt and reshape of the waste plastics have achieved limited success because of the high cost associated with collecting and sorting processes and the relative inferior properties of the recycled materials to virgin plastics, and thus only 9% of all produced plastics have been recycled.^[^ ^5^ ^]^ Therefore, it is pivotally important to develop alternatively efficient and cost‐effective techniques to recycle these naturally non‐degradable waste plastics.

**Figure 1:**
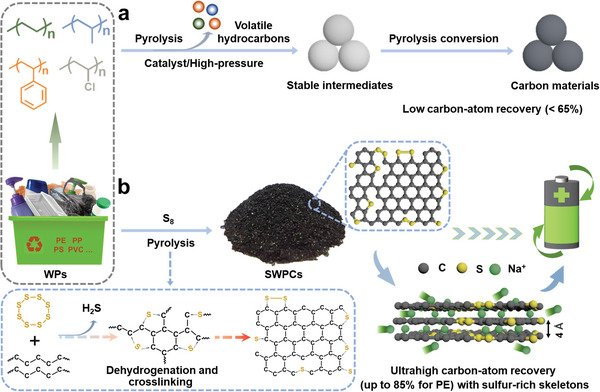
Schematic illustration of different strategies for upcycling of waste plastics (WPs) into high‐value carbon materials, including a) the conventional pyrolysis strategy with catalysts and/or high‐pressure equipment and b) our pyrolysis strategy with the presence of inexpensive elemental sulfur. The conventional pyrolysis strategy brings additional environmental and economic concerns and suffers a low carbon‐atom recovery. In sharp contrast, our sulfur‐assisted pyrolysis strategy is simple and efficient with an ultrahigh carbon‐atom recovery. Note that in a possible sulfur‐assisted prolysis mechanism in (b), PE is selected as an example of waste plastics, and hydrogen atoms in its chemical structure are not provided for simplicity.

Over the past decades, great efforts have been devoted to chemical upcycling of the plastics by using them as sustainable resources for the synthesis of high value‐added products or the recovery of monomers.^[^ ^6^ ^]^ Chemical (pyrolytic or catalytic) or biological (enzymatic) depolymerization is such an important approach to upcycle the waste plastics into more valuable small molecules that are usually used as chemical feedstocks, fuels, and lubricants.^[^ ^7^ ^]^ Nevertheless, this strategy often generates complex mixtures of gases, liquid hydrocarbons, and chars, and thus additional energy‐intensive refinement is needed to recover these outputs for further use. Meanwhile, slow depolymerization rates, low yields of higher‐value products, and intolerance to real‐world mixed waste plastics greatly hamper the commercial application. Recently, considering the abundance of carbon atoms in plastics, pyrolyzing waste plastics into high‐value carbon materials has attracted increasing attention due to its high tolerance to the chemical heterogeneity of waste plastics and wide applications of the resulting carbon materials in various areas.^[^ ^8^ ^]^ Generally, pyrolysis of plastics involves two competing reactions, including thermal degradation into volatile small hydrocarbons and thermal crosslinking into stable polymeric intermediates to produce carbon materials, respectively. Due to the dominantly thermal degradation process, most of the waste plastics such as PE, PP, and PS are completely decomposed into volatile small hydrocarbons upon direct pyrolysis, with negligible carbonaceous residues. In this context, additional catalysts and/or high‐pressure equipment are developed to facilitate the formation of thermally stable polymeric intermediates, and thus promote the pyrolysis conversion of these plastics into carbon materials (**Figure** **1**a).^[^ ^9^ ^]^ Nevertheless, the tedious procedures resulting from subsequent removal of catalysts as well as the harsh pyrolysis conditions bring additional environmental and economic concerns. More importantly, these pyrolysis strategies remain suffering a low carbon‐atom recovery (defined as the ratio of the carbon atoms preserved after pyrolysis, typically < 65%, Table S1, Supporting Information), thereby leading to not only a large waste of resources but also high greenhouse gas emissions. Hence, it is highly desirable from both the environmental and economic points of view to develop facile solutions for high‐atom‐economy conversion of waste plastics into high‐value carbon materials.

Herein, for the first time, a sulfur‐assisted pyrolysis strategy is developed for simple, effective, and versatile upcycling of the real‐world waste plastics into high‐value carbons with an ultrahigh carbon‐atom recovery (Figure 1b). This strategy involves the pyrolysis of waste plastics with the presence of inexpensive elemental sulfur in a conventional tubular furnace, which does not need harsh conditions and complex procedures and is therefore easy to scale up. During the first stage of pyrolysis process, the elemental sulfur molecules undergo ring‐opening polymerization into a linear polysulfane with diradical chain ends, which can attack the carbon‐hydrogen bonds and chemically crosslink the plastics to produce thermally stable intermediate polymeric structures. As a result, the subsequent decomposition of plastics into volatile small hydrocarbons at higher temperatures is greatly inhibited, which enables the successful upcycling of plastics into high‐value carbon materials with an unprecedented carbon‐atom recovery (up to 85% for PE). The as‐obtained carbon products (sulfur‐rich waste plastic‐derived carbons, SWPCs) have sulfur‐rich skeletons with an enlarged interlayer distance, and thus demonstrate great application potential for high‐performance sodium‐ion batteries (SIBs). This work offers a promising strategy to alleviate plastic pollution and reduce the carbon footprint of plastic industry.

## 2 Results and Discussion

### 2.1 Mechanism of Sulfur‐Assisted Pyrolysis

Elemental sulfur is mainly obtained as a by‐product in the hydrodesulfurization process of industrial petroleum refining. The sheer abundance and low cost of elemental sulfur brings a strong motivation to utilize it as a feedstock for the synthesis of novel functional materials. It is well known that elemental sulfur exists primarily in the form of cyclic molecule with eight sulfur atoms at ambient conditions, and linear polysulfane with diradical chain ends can be formed upon heating up to 159 °C because of the ring‐opening polymerization.^[^ ^10^ ^]^ As such, elemental sulfur can be easily incorporated into polymer materials to modify their properties.^[^ ^11^ ^]^ Inspired by this, we propose to employ elemental sulfur to facilitate the conversion of waste plastics into high‐value carbons.

**Figure 2:**
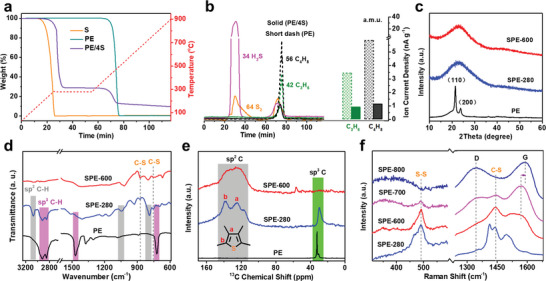
a) TGA curves of S, PE, and PE/4S, and b) corresponding TGA‐MS spectra of PE and PE/4S tested in N_2_ with a heating rate of 10 °C min^−1^ and preservation stage of 280 °C for 30 min. c) XRD, d) FT‐IR, and e) ^13^C‐NMR spectra of PE, SPE‐280, and SPE‐600. f) Raman spectra of SPE‐280, SPE‐600, SPE‐700, and SPE‐800.

The thermal decomposition of plastics with or without elemental sulfur is first investigated by thermogravimetric analysis (TGA). As expected, both of the individual elemental sulfur and polyolefins including PE, PP, and PS present one weight‐loss peak, and are completely decomposed under N_2_ atmosphere (**Figure** **2**a and Figure S1, Supporting Information). Notably, the elemental sulfur can effectively assist the thermal transformation of polyolefins into carbon materials. Taking PE as a typical example, although two weight‐loss peaks are observed for the mixture of PE and elemental sulfur (1:4 by weight, PE/4S), a considerable solid residue of 13 wt% is retained under 900 °C, indicating the successful formation of carbon materials. TGA coupled with mass spectroscopy (TGA‐MS) in Figure 2b reveals that a large amount of H_2_S is released for the mixture of PE/4S during the pyrolysis process, suggestive of obvious dehydrogenation reactions between PE and in situ formed linear polysulfanes with diradical chain ends. This dehydrogenation process enables the inter‐ and intramolecular crosslinking of PE chains with polysulfane bridges that can be further decomposed into volatile sulfur segments (e.g., S_2_). Notably, the polymeric intermediate derived from PE/4S mixture demonstrates greatly enhanced thermal stability, as evidenced by the much fewer released volatile hydrocarbon emissions (typically C_3_H_6_ and C_4_H_8_) than the pure PE at high temperatures.

To further track the sulfur‐assisted pyrolysis behavior of polyolefins, the structural characteristics of the PE/4S mixtures treated at different temperatures (SPE‐*T*, *T* represents the treating temperature) are also investigated. As shown in X‐ray diffraction (XRD) spectra (Figure 2c and Figure S2, Supporting Information), both PE and its product treated at 280 °C for 4 h (PE‐280) have two obvious diffraction peaks at 21.5° and 23.8° corresponding to (110) and (200) planes of the typical orthorhombic PE crystals, respectively,^[^ ^12^ ^]^ indicating that such a treatment without sulfur does not change the crystalline structure of PE. However, for SPE‐280, all of the crystal peaks of PE and S disappear and only a broad peak of amorphous structure is observed,^[^ ^13^ ^]^ suggestive of markedly changed molecular structure of PE chains after reaction with elemental sulfur. As shown in Fourier‐transform infrared (FT‐IR) spectra of Figure 2d, the absorption peaks related to saturated —CH_2_— groups (e.g., 2920, 2850, 1467, and 721 cm^−1^) of PE are vanished or weakened after sulfurization at 280 °C, accompanied by the appearance of a series of new characteristic peaks of unsaturated sp^2^ C—H in thiophene rings (e.g., 3070, 1045, and 789 cm^−1^) and C─S bonds (e.g., 754 and 875 cm^−1^) for SEP‐280.^[^ ^14^ ^]^ The solid‐state ^13^C nuclear magnetic resonance (NMR) spectrum of SPE‐280 further confirms the presence of conjugated thiophene rings with sp^2^ carbons, as verified by the two characteristic peaks at 124.5 and 138.8 ppm (Figure 2e).^[^ ^14c^ ^]^ In addition to the symmetric in‐phase vibration of thiophene rings (1440 cm^−1^), a sharp band located at 487 cm^−1^ is also observed in the Raman spectrum of SPE‐280, which can be ascribed to stretching vibration of S—S bonds (Figure 2f).^[^ ^15^ ^]^ The results clearly indicate that the sulfurization process can dehydrogenize most of the —CH_2_— groups in PE, which is beneficial for crosslinking the linear polymeric chains to produce thermally stable crosslinked polymeric intermediates with abundant sulfur‐containing groups (Figure S3 and Table S2, Supporting Information). As shown in Figure S4, Supporting Information, the SPE‐280 can retain a carbonization yield of over 50% when heating to 900 °C in N_2_ atmosphere, suggesting the key role of sulfurization process for sulfur‐assisted thermal conversion of polyolefins into carbon materials. The inevitable mass loss is mainly ascribed to the decomposition of remaining —CH_2_─ groups and in situ formed S—S species at higher temperatures, as verified by the FT‐IR, ^13^C NMR, and Raman spectra of the PE/4S mixtures treated at higher temperatures, which is consistent with the TGA‐MS results (Figure 2b–f). It should be noted that the G‐band, corresponding to the in‐plane vibration of microcrystalline graphite, is upshifted from 1568 cm^−1^ for SPE‐600 and SPE‐700 to 1586 cm^−1^ for SPE‐800 (Figure 2f). The results demonstrate that the remaining sulfur atoms in SPE‐600 and SPE‐700 are beneficial for increasing the interlayer spacing of microcrystalline graphite.^[^ ^16^ ^]^

**Figure 3:**
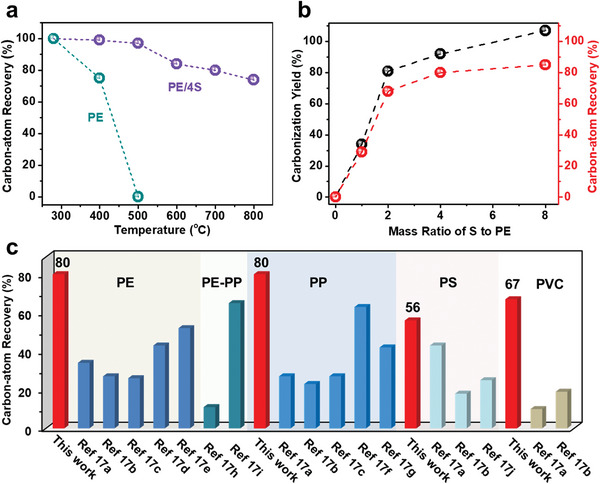
The carbon‐atom recovery of a) PE and PE/4S treated at various temperatures. b) The carbonization yield and carbon‐atom recovery of the S and PE mixtures with various feed ratios treated at 700 °C. c) Comparison of carbon‐atom recovery between our strategy and other reported methods.

In view of the high sulfur contents in polyolefin‐derived carbon materials, carbon‐atom recovery is further carefully investigated to clarify the upcycling efficiency of the sulfur‐assisted pyrolysis strategy. As consistent with the above results, the carbon‐atom recovery of pure PE is 0% at 500 °C (**Figure** **3**a and Table S2, Supporting Information). In sharp contrast, up to 97% carbon atoms are reserved for the PE/4S mixture before 500 °C, and the carbon‐atom recoveries of SPE‐600, SPE‐700, and SPE‐800 are calculated to be up to 84%, 80%, and 76%, respectively. The content of elemental sulfur also significantly affects the carbon‐atom recovery. Although the related carbon products at 700 °C exhibit similar carbon and sulfur contents when the mass ratio of S to PE increases from 1:1 to 8:1, their yields based on the mass of PE increase significantly from 34% to 107% (Figure 3b and Figure S5, Supporting Information). Correspondingly, the carbon‐atom recovery of the products increases with the S ratio from 29% for 1:1 to 85% for 8:1. The results indicate that the elemental composition of the related carbon products is independent of the S ratio, and increasing the addition amount of elemental sulfur is beneficial for decreasing the ratio of thermally unstable —CH_2_— groups in the polymeric intermediates, thereby leading to a higher carbon‐atom recovery. Notably, the highly efficient recovery of carbon atoms by the sulfur‐assisted pyrolysis strategy is also verified for other naturally non‐degradable polyolefins, as demonstrated by 80% for PP, 67% for PVC, and 56% for PS under similar conditions (Figure 3c). The relatively low carbon‐atom recovery of PS as compared to PE, PP, and PVC resins without benzene rings may result from the relatively low sulfur‐based crosslinking efficiency of its benzene rings (Figure S6, Supporting Information).^[^ ^14a^ ^]^ To the best of our knowledge, thermal conversion of these plastics into carbon materials with such an ultrahigh carbon‐atom recovery has not been reported so far, confirming that the versatile sulfur‐assisted pyrolysis strategy is adoptable and efficient for dealing with non‐charring polymers (Table S1, Supporting Information).^[^ ^17^ ^]^

### 2.2 Converting Real‐World Waste Plastics into Sulfur‐Doped Carbons

**Figure 4:**
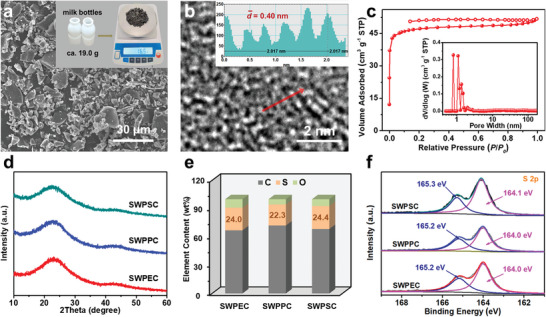
a) SEM image, b) HRTEM image, and c) N_2_ adsorption–desorption isotherm of SWPEC. d) XRD spectra, e) element content, and f) high‐resolution S 2p XPS spectra of SWPEC, SWPPC, and SWPSC. The inset in (a) is digital photo of wasted milk bottles and their derived carbon product; the inset in (b) is corresponding intensity profile for the line scan across the lattice fringes; the inset in (c) is corresponding pore size distribution curve.

To demonstrate the potential application and feasibility of the sulfur‐assisted pyrolysis strategy for upcycling real‐world waste plastics into high‐value carbons, three typical waste plastics (e.g., milk bottles, disposable food boxes, and vessels) collected from our daily life wastes are mixed with elemental sulfur (1:4 by mass) and thermally treated under the same conditions as SPE‐700. The carbon‐atom recoveries of SWPCs are close to those of counterparts prepared from pure resins, indicating similar chemical reactions during the pyrolysis process (Table S3, Supporting Information). It should be noted that the escaped sulfur species in the form of sublimated sulfur and gaseous H_2_S can be easily recycled in a relatively closed system (Figure S7, Supporting Information). As shown in the inset digital photo of **Figure** **4**a, 19.0 g of waste milk bottles (made of PE) can produce 16.5 g of carbons (SWPEC) after sulfur‐assisted pyrolysis process, indicating that the sulfur‐assisted pyrolysis strategy is able to meet the demand of large‐scale application. Scanning electron microscopy (SEM) image reveals that the SWPEC is in the form of irregular particles (Figure 4a). Element mapping analysis of SWPEC demonstrates the presence and homogeneous distribution of C and S elements (Figure S8, Supporting Information). High‐resolution transmission electron microscopy (HRTEM) image shows that the SWPEC is composed of disorderly packed turbostratic carbon nanodomains with an enlarged interlayer distance of 0.4 nm (Figure 4b). The disordered packing of these turbostratic carbon nanodomains can result in free volume and microporosity in the carbon skeleton. As confirmed by nitrogen adsorption/desorption test, the Brunauer–Emmett–Teller surface area (*S*_BET_) of SWPEC is measured to be 157 m^2^ g^−1^ with the pore sizes centered below 2 nm, indicating a predominate microporous structure (Figure 4c). Notably, although the molecular weights and structures of wasted disposable food boxes (made of PP) and vessels (made of PS) are different from those of milk bottles, both of their upcycled carbon products, denoted as SWPPC and SWPSC, respectively, exhibit similar morphologies and structures to SWPEC, suggesting great versatility of the sulfur‐assisted upcycling strategy (Figures S9 and S10 and Table S4, Supporting Information).

The XRD spectra in Figure 4d show that all of the SWPEC, SWPPC and SWPSC exhibit a broad and weak (002) diffraction peak centered at 22.3° with a *d*‐spacing of 0.40 nm, consistent with the HRTEM results. The enlarged interlayer spacing can enhance the ionic intercalation and deintercalation kinetics, which is beneficial for high‐performance alkali‐ion batteries.^[^ ^18^ ^]^ The chemical compositions of the three products are further investigated by X‐ray photoelectron spectroscopy and reveal the presence of C, S, and O elements (Figure S11, Supporting Information). The sulfur content is calculated to be as high as 24.0 wt% for SWPEC, 22.3 wt% for SWPPC, and 24.4 wt% for SWPSC (Figure 4e and Table S5, Supporting Information). The efficient sulfur doping can manipulate the electronic structures of the carbon skeleton for enhanced performance in emerging energy storage and conversion technologies.^[^ ^19^ ^]^ The results are similar to that of elemental analysis (EA), demonstrating that sulfur is uniformly bonded into carbon skeleton (Table S3, Supporting Information). The two peaks in the high‐resolution S 2p spectra at 164.0 and 165.2 eV can be assigned to the S 2p_3/2_ and 2p_1/2_ peaks of S—S/C—S bonds (Figure 4f).^[^ ^20^ ^]^

### 2.3 Sodium Storage Performance of Sulfur‐Rich Waste Plastic‐Derived Carbons

In recent years, SIBs have been regarded as one of the emerging alternatives to the market‐dominant lithium‐ion batteries due to the abundance and low cost of sodium resource. Developing high‐performance anode materials with high capacity, favorable capability, and excellent stability is a key requirement to realize the commercialization of SIBs.^[^ ^21^ ^]^ Benefiting from high reversible charge storage capacity and favorable ionic diffusion kinetics during the discharge/charge process, heteroatom‐doped carbon materials have recently attracted great attention.^[^ ^22^ ^]^ In this regard, the carbon materials derived from waste plastics will be very promising anode materials for SIBs because of their sulfur‐rich skeletons with enlarged interlayer distance. The sodium storage performances of the SWPCs are investigated by cyclic voltammogram (CV) and galvanostatic charge–discharge measurements in half cell tests. As shown in Figure S12, Supporting Information, the SWPCs exhibit similar CV curves in the range of 0.01–3 V (vs Na/Na^+^) at a scan rate of 0.1 mV s^−1^, suggestive of identical structures. In the first scan, the broad reduction peak below 0.6 V can be attributed to the formation of the solid electrolyte interphase, and one pair of redox peaks at 1.80/1.08 V correspond to the redox reactions between Na^+^ and S species.^[^ ^23^ ^]^ In the following scans, the CV curves are almost overlapped, suggesting good reversibility of the SWPCs. Figure S13, Supporting Information, displays the discharge−charge profiles of the SWPCs in the first five cycles at a current density of 0.05 A g^−1^. The plateau voltage in the profiles is coincident with the peak location in the CV curves, and the sloping platform reveals a capacitive‐dominated Na^+^ storage behavior. Notably, high reversible capacities of up to 662 mAh g^−1^ for SWPEC, 578 mAh g^−1^ for SWPPC, 661 mAh g^−1^ for SWPSC are achieved in the first cycle, much higher than that of conventional hard carbon materials (typically ≤ 300 mAh g^−1^).

**Figure 5:**
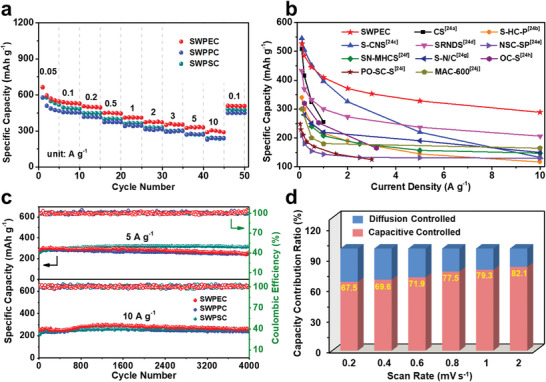
a) Rate performance of SWPEC, SWPPC, and SWPSC. b) Rate performance comparison between SWPEC and reported heteroatom‐doped carbonaceous anodes. c) Cycling performance of SWPEC, SWPPC, and SWPSC. d) The contribution ratio of capacitive and diffusion controlled capacity of SWPEC at different scan rates.

Benefiting from the unique structure, SWPCs also exhibit an outstanding cycling response to continuously varied current densities. When cycled at current densities of 0.1–5 A g^−1^, favorable reversible capacities of 328–526 mAh g^−1^ are obtained for SWPEC (**Figure** **5**a). Importantly, a capacity as high as 289 mAh g^−1^ can be achieved even when operating at an ultrahigh current density of 10 A g^−1^, suggestive of highly efficient kinetics for Na storage. After abruptly switching the current density back to 0.1 A g^−1^, high capacity of 509 mAh g^−1^ could be largely restored for repeated stable cycling, indicating the excellent robustness and stability of SWPEC. Owing to their identical structures, SWPPC and SWPSC also exhibit excellent rate performances, as demonstrated by 239 mAh g^−1^ for SWPPC and 236 mAh g^−1^ for SWPSC at 10 A g^−1^, respectively. To the best of our knowledge, such high‐rate performances of SWPCs are superior to those of most heteroatom‐doped carbonaceous anode materials reported previously (Figure 5b and Table S6, Supporting Information).^[^ ^24^ ^]^ Long‐term cycling tests reveal that the SWPCs also exhibit excellent durability at various current densities (Figure 5c). For example, the SWPSC delivers a high capacity of 312 mAh g^−1^ at a current rate of 5 A g^−1^ after 4000 cycles. Remarkably, high capacity of 256 mAh g^−1^ could be retained for up to 4000 cycles at a very high current density of 10 A g^−1^ with almost 100% Coulombic efficiency, indicative of highly promising application as anodes of high‐rate SIBs.

To get better understanding of the Na^+^ storage mechanism of SWPCs, the CV curves of SWPEC at various scan rates of 0.2, 0.4, 0.6, 0.8, 1, and 2 mV s^−1^ are recorded (Figure S14a, Supporting Information). The capacity of carbon anodes is contributed by the diffusion‐controlled intercalated charge and the surface‐controlled capacitive charge. According to pervious reports, the capacity can be qualitatively analyzed by the *b*‐value following the equation of

(1) $$i=av^{b}$$

where *i* is the peak current, *v* is the scan rate, and *b* is adjustable value that equals to 0.5 for intercalated process and 1 for capacitive process.^[^ ^25^ ^]^ As shown in Figure S14b, Supporting Information, the *b* value of SWPEC is determined to be 0.99 and 0.87 for the reduction and oxidation state, respectively, indicative of a dominated capacitance process. The proportion of capacitive contributions can be further quantitatively evaluated by

(2) $$i=k_{1}v+k_{2}v^{1/2}$$

where the *k*_1_ *v* and *k*_2_ *v* ^1/2^ represents the diffusion and capacitive behavior contribution to the total capacity, respectively.^[^ ^26^ ^]^ As shown in Figure 5d and Figure S14c, Supporting Information, the contribution of capacitive capacity is promoted with the increment of scan rates from 0.2 to 2 mV s^−1^ and 79.3% of the total capacity is identified as the capacitive contribution at 1 mV s^−1^. The leading capacitive charge storage results from the sulfur‐rich carbon skeletons of SWPCs with an enlarged interlayer distance, which enables Faradaic reactions with fast kinetics and facilitates solid‐state ionic diffusion between the interlayers. It should be noted that comparable carbon‐atom recovery and performance can also be achieved for the product (SWMC) obtained with a mixture of waste plastics PE, PP, and PS (mass ratio of 1:1:1) as precursor, indicating that sulfur‐assisted pyrolysis strategy is an adaptive and reliable approach for upcycling waste plastics into high‐value carbons for SIBs (Table S3 and Figure S15, Supporting Information).

## 3 Conclusion

In summary, a simple, effective, and versatile strategy of sulfur‐assisted thermal conversion has been demonstrated to upcycle waste plastics into sulfur‐rich carbons with an ultrahigh carbon‐atom recovery. It has been demonstrated that elemental sulfur can facilitate the chemical crosslinking of liner polymeric chains to produce thermally stable crosslinked polymeric structures, thereby inhibiting the decomposition of plastics into volatile small hydrocarbons. The as‐obtained carbons exhibit sulfur‐rich skeletons with an enlarged interlayer distance, and thus demonstrate superior sodium storage performance when used as anode materials of SIBs. We believe that the present results offer a new solution to alleviate plastic pollution and reduce the carbon footprint of plastic industry.

## Conflict of Interest

The authors declare no conflict of interest.

## Supporting information

Supporting Information

## Data Availability

The data that support the findings of this study are available from the corresponding author upon reasonable request.
