# Supplementary material for: A Versatile Sulfur‐Assisted Pyrolysis Strategy for High‐Atom‐Economy Upcycling of Waste Plastics into High‐Value Carbon Materials
Source: Adv Sci (Weinh). 2023 Mar 29;10(15):2206924. doi: 10.1002/advs.202206924 (PMC10214230; doi:10.1002/advs.202206924)
Supplement: Supplementary file 1 — Supporting Information [file ADVS-10-2206924-s001.pdf]

Supporting Information

A versatile sulfur-assisted pyrolysis strategy for high-atom-economy upcycling of waste plastics into high-value carbon materials

Youchen Tang[1, 2], Zongheng Cen[2], Qian Ma[3], Bingna Zheng[1], Zhaopeng Cai[1, *], Shaohong Liu[2, *] and Dingcai Wu[2, *]

[1]*Department of Orthopedics, The Eighth Affiliated Hospital, Sun Yat-sen University, Shenzhen 518000, P.R. China.*

[2]*PCFM Lab, School of Chemistry, Sun Yat-sen University, Guangzhou 510006, P. R. China.*

[3]*Research Center of Medical Sciences, Guangdong Provincial People's Hospital, Guangdong Academy of Medical Sciences, Guangzhou 510080, P. R. China.*

**Email: caizhp3@mail.sysu.edu.cn; liushh27@mail.sysu.edu.cn; wudc@mail.sysu.edu.cn*

Experimental Section

Preparation of Samples. The waste plastics were obtained from a supermarket in local campus (Guangzhou), which were washed and granulated into powders. Other chemicals were used directly without extra treatment. SPE-280 were prepared by mixing 0.5 g of PE powder (Alfa Aesar, low density, average molecular weight 44000) with 2 g of S powder (Aladdin, sublimed sulfur), followed by heating at 280 ^{o}C for 4 h with a heating rate of 5 ^{o}C min^{-1}. For a typical preparation of other SPE-*T* (*T* represents the heating temperature except for 280 ^{o}C), a mixture of PE powder (0.5 g) and S powder (2 g) were heated at 280 ^{o}C for 4 h, followed by heating at *T* ^{o}C for 3 h with a heating rate of 5 ^{o}C min^{-1}. 1SPE-700, 2SPE-700, and 8SPE-700 were obtained by changing the mass of S powder to 0.5, 1, and 4 g, respectively. Similarly, SPP-700, SPVC-700, and SPS-700 were prepared with PP (Macklin, melt index 4 g/10 min), PVC (Macklin, K-value 68-65), and PS (Sigma-Aldrich, average molecular weight 192000) powders as precursors, respectively. With waste plastics of PE, PP, PS, and their mixture (mass ratio of 1:1:1) as precursors, SWPEC, SWPPC, SWPSC, and SWMC were obtained, respectively, via the same preparation conditions as SPE-700.

Characterization. Material morphologies was investigated with a field-emission scanning electron microscopy (Hitachi S-4800). Element mapping images were provided by transmission electron microscopy (Tecnai G2 Spirit) at an accelerating voltage of 200 kV. TGA was measured on a TA Instruments Q50 at a heating rate of 10 ^{o}C min^{-1} under N_2 flow. The element composition of the samples was examined by a vario EL cube Elemental Analyzer with accuracy of 0.3% (CHNS/O, Elementar Germany). XPS spectra were obtained on a Thermo SCIENTIFIC ESCALAB 250Xi. FT-IR spectra were provided by the Bruker Equinox 55. Magic-angle spinning solid-state NMR spectra were acquired on a 600 MHz Bruker Neo spectrometer using a 1.6 mm HFX probe. Raman spectra were obtained on an inVia RENISHAW with 514 nm lasers. XRD spectra were collected on a RIGAKU D-MAX 2200 VPC with Cu-Kα radiation. The N_2 adsorption-desorption test was conducted on a Micromeritics ASAP 2020 analyser at 77 K.

Electrochemical Measurements. The electrochemical measurements of the as-obtained carbon products were conducted at room temperature by using CR2025 coin cells with pure Na foil as counter electrode, 1 M $NaClO_4$ in ethyl carbonate (EC)/propylene carbonate (PC) (1:1 by volume) with 5 vol% of fluoroethylene carbonate (FEC) as electrolyte, and Whatman glass membrane (GF/D) as separator. The working electrodes consisted of carbon products, Super P, and polyvinyl difluoride (PVDF) in a mass ratio of 7:2:1. The mass loading of carbon products in the electrodes was around 1 mg cm^{-2}. The CV tests were measured on a CHI660e electrochemical workstation (Shanghai Chenhua). The galvanostatic charge-discharge tests were recorded on Wuhan LAND battery testers in the voltage range of 0.01-3 V.

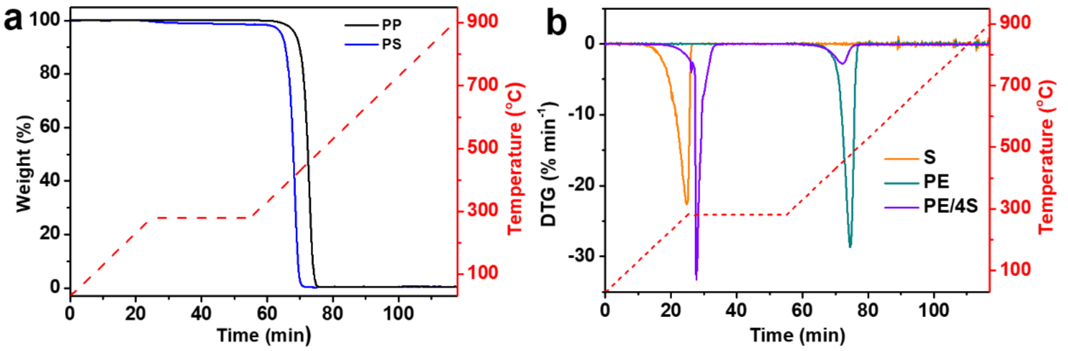

Figure S1 (a) TGA curves of PP and PS, and (b) DTG curves of S, PE and PE/4S tested in N_2 with a heating rate of 10 °C min^{-1} and preservation stage of 280 °C for 30 min.

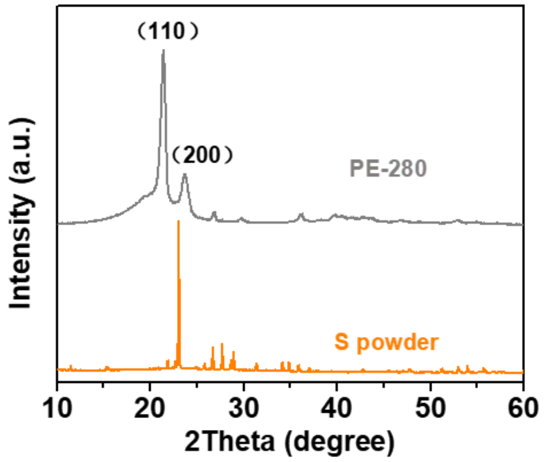

Figure S2 XRD spectra of elemental sulfur powder and PE-280.

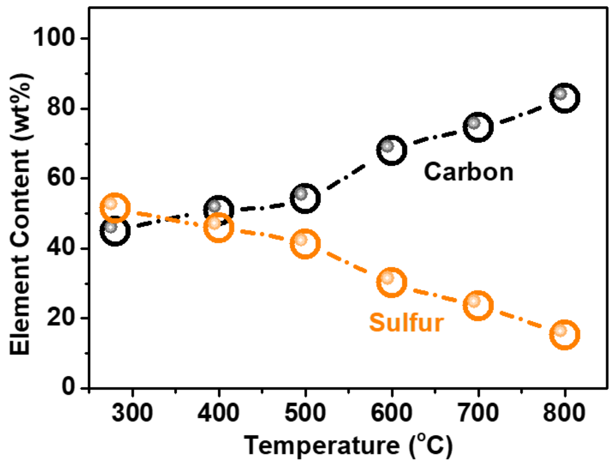

Figure S3 The carbon and sulfur contents of PE/4S treated at various temperatures.

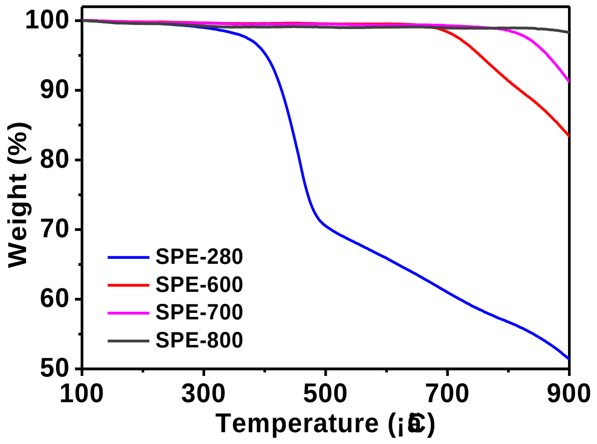

Figure S4 TGA curves of SPE-280, SPE-600, SPE-700, and SPE-800 tested in N_2 with a heating rate of 10 °C min^{-1}.

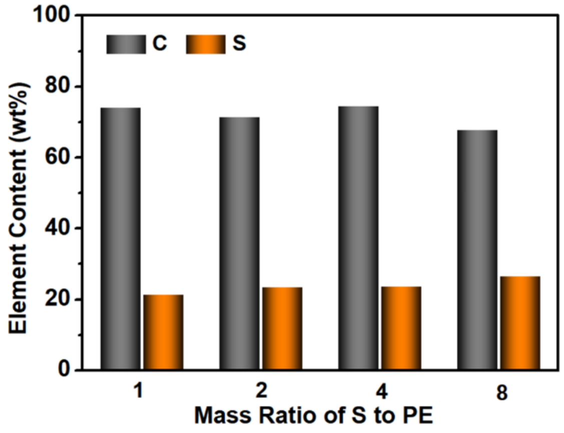

Figure S5 The carbon and sulfur contents of the carbon products obtained by treating at 700 °C with different feed ratios.

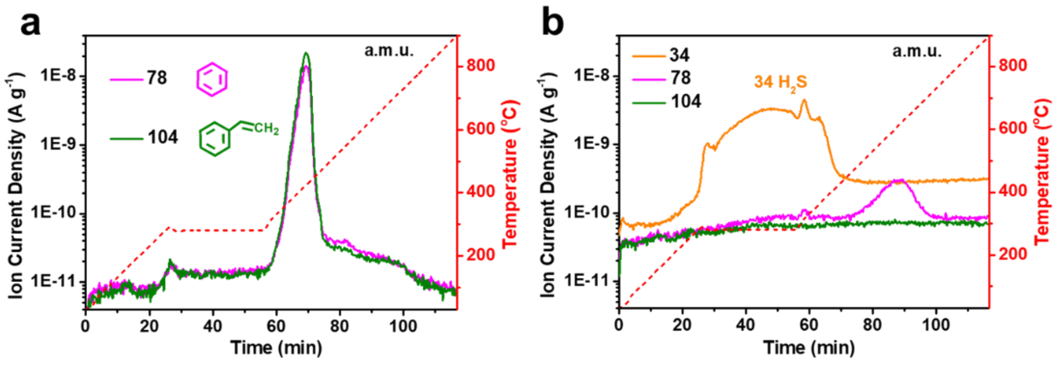

Figure S6. TGA-MS spectra of (a) PS and (b) PS/4S with a heating rate of 10 °C min^{-1} and preservation stage of 280 °C for 30 min. A peak around 600 °C is observed, which may correspond to the loss of benzene ring species, resulting in a relatively low carbon-atom recovery of 56%.

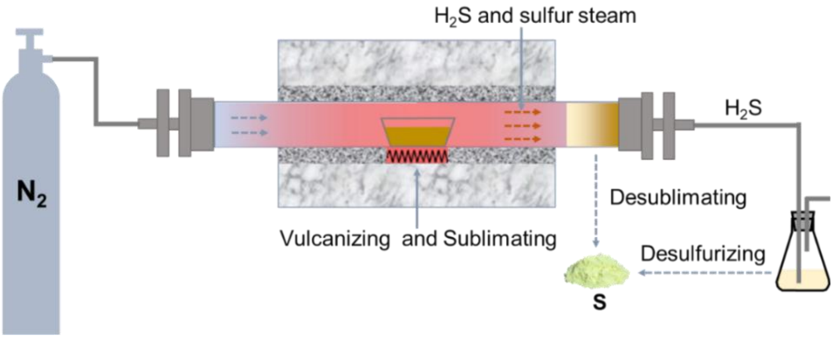

Figure S7 Schematic diagram showing the recycling process of elemental sulfur. In a relatively closed system, S can be easily recycled from the sublimated sulfur and generated H_2S, because that sulfur steam desublimates at the end of furnace tube with lower temperature and H_2S can be reduced into S by desulfurization reagents (e.g., sodium thiosulfate).

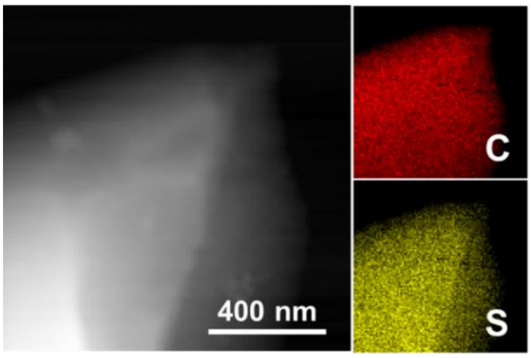

Figure S8 Element mapping images of SWPEC.

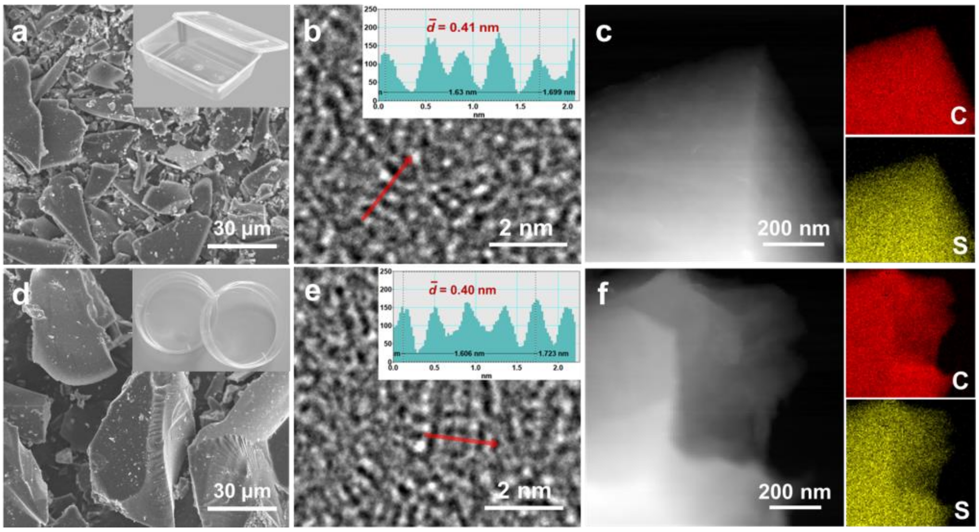

Figure S9 (a,d) SEM, (b,e) HRTEM, and (c,f) element mapping images of (a-c) SWPPC and (d-f) SWPSC. The insets in (a,d) are digital photos of the related precursors, and the inset in (b,e) are corresponding intensity profiles for the arrow scan across the lattice fringes.

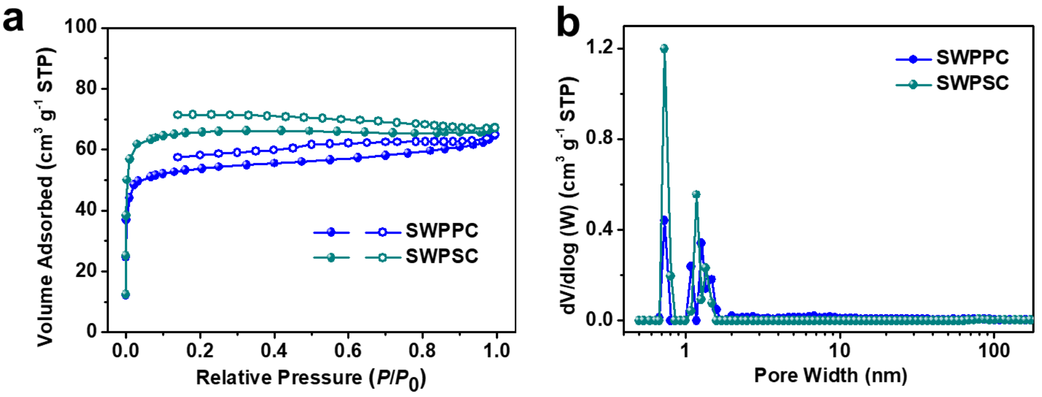

Figure S10 (a) N_2 adsorption-desorption isotherms and (b) pore size distribution curves of SWPPC and SWPSC.

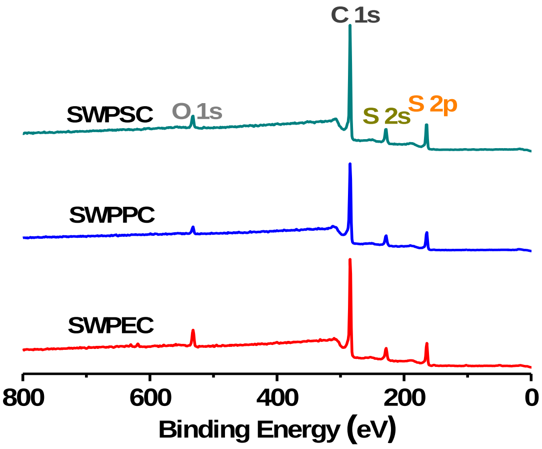

Figure S11 XPS full scan spectra of SWPEC, SWPPC and SWPSC.

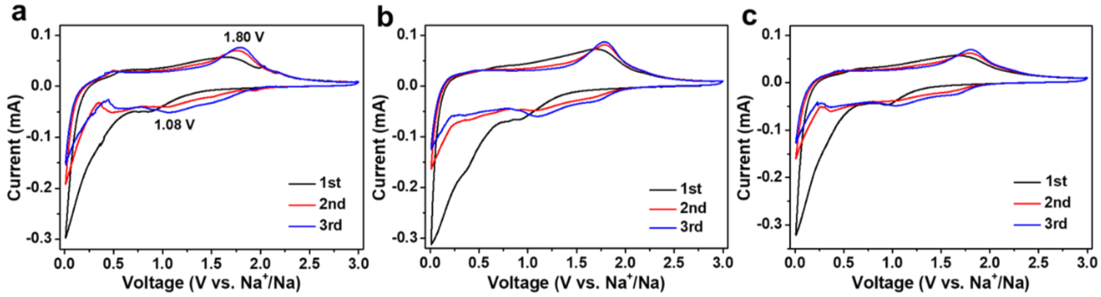

Figure S12 CV curves of (a) SWPEC, (b) SWPPC, and (c) SWPSC in the range of 0.01-3 V (vs Na^+/Na) at a scan rate of 0.1 mV s^{-1}.

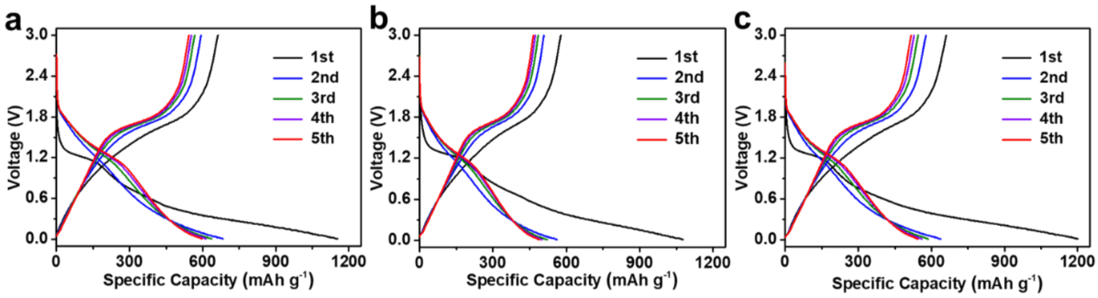

Figure S13 The initial five galvanostatic charge-discharge curves of (a) SWPEC, (b) SWPPC, and (c) SWPSC at 0.05 A g^{-1}.

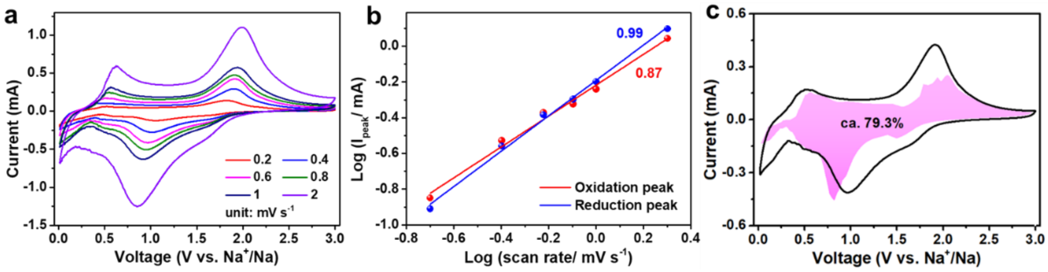

Figure S14 (a) CV curves of SWPEC at various scan rates ranging from 0.2 to 1 mV s^{-1}. (b) Relationship between the peak current and scan rate in logarithmic format. (c) Capacitive contribution (pink) to charge storage at a scan rate of 1 mV s^{-1}.

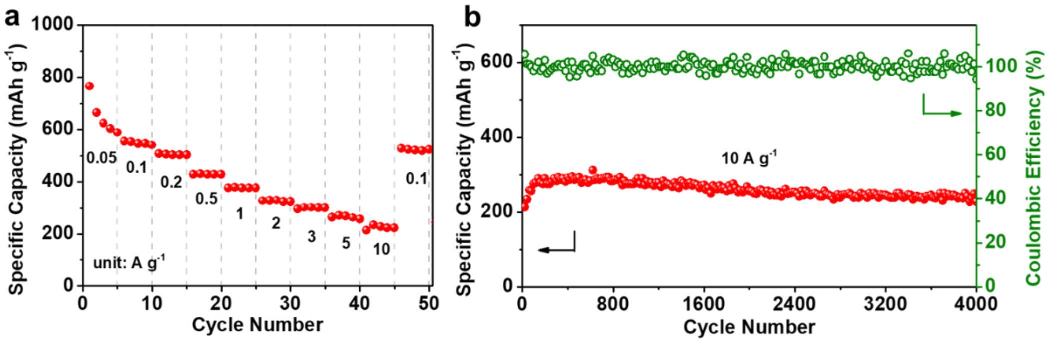

Figure S15 (a) Rate and (b) cycling performance of SWMC obtained with a mixture of waste PE, PP, and PS (mass ratio of 1:1:1) as precursor.

Table S1. The carbon-atom recovery comparison of the carbon products obtained from (waste) plastics via different methods.

Precursors	Method	Temperature (ºC)	Carbon-atom recovery (%)	References
**PE/PP/PS/PVC**	**Sulfur-assisted pyrolysis**	**700**	**80/80/56/67**	**This work**
PE/PP/PS/PVC	Chemical vapor deposition	--	34/27/43/10	*Nanoscale* **2017**, *9*, 9089
PE/PP/PS/PVC	Flash Joule heating	--	27/23/18/19	*ACS Nano* **2020**, *14*, 15595
PE/PP	Catalytic pyrolysis	700	26/27	*J. Clean. Prod.* **2020**, *258*, 120633
PE	Flash Joule heating	--	43	*ACS Nano* **2022**, *16*, 7804
PE	High-pressure pyrolysis	600	52*	*ACS Sustainable Chem. Eng.* **2019**, *7*, 3801
PP	Catalytic pyrolysis	700	63	*Carbon* **2021**, *171*, 819
PP	Catalytic pyrolysis	800	42*	*Chem. Eng. J.* **2021**, *408*, 127268
PE-PP	Catalytic pyrolysis	600	11	*ACS Nano* **2022**, *16*, 7284
PE-PP	Catalytic pyrolysis	800	65*	*Appl. Catal. B: Environ.* **2021**, *280*, 119413
PS	Catalytic pressure pyrolysis	700	25	*Waste Manage.* **2019**, *85*, 333

*The carbon contents of the carbon products are assumed to be 100%.

Table S2. Carbonization yield, element content, and carbon-atom recovery of different samples.

Samples	Carbonization yield (%)	C (wt%)	S (wt%)	H (wt%)	Carbon-atom recovery (%)
PE-400	75	86.1	--	13.9	75
PE-500	0	--	--	--	0
SPE-280	191	44.8	51.5	1.8	100
SPE-400	167	50.8	45.9	--	99
SPE-500	153	54.3	41.1	--	97
SPE-600	106	68.0	30.2	--	84
SPE-700	92	74.5	23.7	--	80
SPE-800	76	82.9	15.2	--	74
1SPE-700	34	74.1	21.5	--	29
2SPE-700	81	71.5	23.5	--	68
8SPE-700	107	67.8	26.6	--	85
SPP-700	94	72.9	18.5	--	80
SPVC-700	39	64.6	29.8	--	67
SPS-700	80	64.1	27.2	--	56

Note: The element content is collected from elemental analysis. Carbonization yield is the mass ratio of the carbon residue to the polymer in the mixture. Carbon-atom recovery is calculated by the formula: carbon-atom recovery (%) = carbonization yield (%) × carbon content of sample (wt%)/theoretical carbon content of precursor.

Table S3. Carbonization yield and carbon-atom recovery of SWPCs.

Samples	C (wt%)	H (wt%)	S (wt%)	Carbonization yield (%)	Carbon-atom recovery (%)
PE milk bottles	85.1	12.6	--	--	--
PP food boxes	84.9	12.9	--	--	--
PS vessels	91.4	7.0	--	--	--
SWPEC	67.5	--	26.5	94	75
SWPPC	68.9	--	22.1	92	75
SWPSC	71.1	--	24.2	76	59
SWMC	63.7	--	26.6	89	65

Note: The content of elements is measured by elemental analysis. Carbon-atom recovery is calculated by the formula: carbon-atom recovery (%) = carbonization yield (%) × carbon content of product (wt%)/carbon content of precursor (wt%).

Table S4. Summary of the pore parameters for SWPCs.

Samples	S_{BET} (m^2 g^{-1})	S_{mic} (m^2 g^{-1})	V_{mic} (cm^3 g^{-1})	V_t (cm^3 g^{-1})
SWPEC	157	132	0.06	0.08
SWPPC	180	143	0.07	0.10
SWPSC	219	192	0.09	0.10

Note: S_{BET}, S_{mic}, V_{mic}, V_t denote BET surface area, micropore surface area, micropore volume, and total pore volume, respectively. The V_t was calculated according to the amount adsorbed at a relative pressure P/P_0 of about 0.99.

Table S5. Element content of SWPCs converted from XPS results.

Samples	C (wt%)	O (wt%)	S (wt%)
SWPEC	67.0	9.0	24.0
SWPPC	72.1	5.6	22.3
SWPSC	68.4	7.2	24.4

Table S6. The electrochemical performances comparison between SWPCs and previously reported heteroatom-doped carbon materials.

Samples	Heteroatoms	Electrochemical performances	References
**SWPEC**	**S**	**289-526 mAh g^{-1} at 0.1-10 A g^{-1}**<br>**256 mAh g^{-1} for 4000 cycles at 10 A g^{-1}**	**This work**
CS	S	255-508 mAh g^{-1} at 0.1-1 A g^{-1}<br>290 mAh g^{-1} for 200 cycles at 1 A g^{-1}	S1
S-HC-P	S	117-340 mAh g^{-1} at 0.1-10 A g^{-1}<br>200 mAh g^{-1} for 4000 cycles at 1 A g^{-1}	S2
S-CNS	S	133-545 mAh g^{-1} at 0.1-10 A g^{-1}<br>211 mAh g^{-1} for 2000 cycles at 5 A g^{-1}	S3
SRNDC	S, N	206-432 mAh g^{-1} at 0.1-10 A g^{-1}<br>161 mAh g^{-1} for 4000 cycles at 10 A g^{-1}	S4
NSC-SP	S, N	130-210 mAh g^{-1} at 0.1-10 A g^{-1}<br>150 mAh g^{-1} for 3200 cycles at 0.5 A g^{-1}	S5
SN-MHCS	S, N	147-240 mAh g^{-1} at 0.5-10 A g^{-1}<br>176 mAh g^{-1} for 2000 cycles at 0.5 A g^{-1}	S6
S-N/C	S, N	150-300 mAh g^{-1} at 0.1-10 A g^{-1}<br>211 mAh g^{-1} for 1000 cycles at 1 A g^{-1}	S7
OC-S	S, O	165-320 mAh g^{-1} at 0.2-3.2 A g^{-1}<br>248 mAh g^{-1} for 4800 cycles at 1 A g^{-1}	S8
PO-SC-S	P, O	125-249 mAh g^{-1} at 0.03-3 A g^{-1}	S9
MAC-600	P, N	165-300 mAh g^{-1} at 0.1-10 A g^{-1}<br>161 mAh g^{-1} for 10000 cycles at 10 A g^{-1}	S10

References

[S1] J. Tzadikov, N. R. Levy, L. Abisdris, R. Cohen, M. Weitman, I. Kaminker, A. Goldbourt, Y. Ein-Eli, M. Shalom, *Adv. Funct. Mater.* **2020**, *30*, 2000592.

[S2] Z. Hong, Y. Zhen, Y. Ruan, M. Kang, K. Zhou, J. M. Zhang, Z. Huang, M. Wei, *Adv. Mater.* **2018**, *30*, 1802035.

[S3] G. Zhao, D. Yu, H. Zhang, F. Sun, J. Li, L. Zhu, L. Sun, M. Yu, F. Besenbacher, Y. Sun, *Nano Energy* **2020**, *67*, 104219.

[S4] B. Yin, S. Liang, D. Yu, B. Cheng, I. L. Egun, J. Lin, X. Xie, H. Shao, H. He, A. Pan, *Adv. Mater.* **2021**, *33*, 2100808.

[S5] D. Xu, C. Chen, J. Xie, B. Zhang, L. Miao, J. Cai, Y. Huang, L. Zhang, *Adv. Energy Mater.* **2016**, *6*, 1501929.

[S6] D. Ni, W. Sun, Z. Wang, Y. Bai, H. Lei, X. Lai, K. Sun, *Adv. Energy Mater.* **2019**, *9*, 1900036.

[S7] J. Yang, X. Zhou, D. Wu, X. Zhao, Z. Zhou, *Adv. Mater.* **2017**, *29*, 1604108.

[S8] T. Wu, M. Jing, Y. Tian, L. Yang, J. Hu, X. Cao, G. Zou, H. Hou, X. Ji, *Adv. Funct. Mater.* **2019**, *29*, 1900941.

[S9] F. Xie, Y. Niu, Q. Zhang, Z. Guo, Z. Hu, Q. Zhou, Z. Xu, Y. Li, R. Yan, Y. Lu, M. M. Titirici, Y. S. Hu, *Angew. Chem. Int. Ed.* **2022**, *61*, 202116394.

[S10] G. Wang, M. Shao, H. Ding, Y. Qi, J. Lian, S. Li, J. Qiu, H. Li, F. Huo, *Angew. Chem. Int. Ed.* **2019**, *58*, 13584-13589.
